# Wnt5a-mediated autophagy contributes to the epithelial-mesenchymal transition of human bronchial epithelial cells during asthma

**DOI:** 10.1186/s10020-024-00862-3

**Published:** 2024-06-19

**Authors:** Yu-Biao Liu, Xiao-Hua Tan, Hui-Hui Yang, Jin-Tong Yang, Chen-Yu Zhang, Ling Jin, Nan-Shi-Yu Yang, Cha-Xiang Guan, Yong Zhou, Shao-Kun Liu, Jian-Bing Xiong

**Affiliations:** 1 Department of Pulmonary and Critical Care Medicine, the Second Xiangya Hospital, Central South University, Changsha, Hunan 410011 China; 2 Department of Physiology, School of Basic Medical Science, Central South University, Changsha, Hunan 410078 China; 3 Key Laboratory of General University of Hunan Province, Basic and Clinic Research in Major Respiratory Disease, Changsha, Hunan 410078 China; 4 Experimental Center of Medical Morphology, School of Basic Medicine Science, Central South University, Changsha, Hunan 410078 China; 5 Department of Emergency, Xiangya Hospital, Central South University, Changsha, Hunan 410078 China; 6 National Clinical Research Center of Geriatric Disorders, Xiangya Hospital, Central South University, Changsha, Hunan 410078 China; 7 Research Unit of Respiratory Disease, Central South University, Changsha, Hunan 410011 China; 8 Clinical Medical Research Center for Pulmonary and Critical Care Medicine in Hunan Province, Changsha, Hunan 410011 China; 9 Diagnosis and Treatment Center of Respiratory Disease, Central South University, Changsha, Hunan 410011 China

**Keywords:** Airway remodeling, Wnt5a, Epithelial-mesenchymal transition, Autophagy, Calmodulin-dependent kinase II, Asthma

## Abstract

**Background:**

The epithelial-mesenchymal transition (EMT) of human bronchial epithelial cells (HBECs) is essential for airway remodeling during asthma. Wnt5a has been implicated in various lung diseases, while its role in the EMT of HBECs during asthma is yet to be determined. This study sought to define whether Wnt5a initiated EMT, leading to airway remodeling through the induction of autophagy in HBECs.

**Methods:**

Microarray analysis was used to investigate the expression change of *WNT5A* in asthma patients. In parallel, EMT models were induced using 16HBE cells by exposing them to house dust mites (HDM) or interleukin-4 (IL-4), and then the expression of Wnt5a was observed. Using in vitro gain- and loss-of-function approaches *via* Wnt5a mimic peptide FOXY5 and Wnt5a inhibitor BOX5, the alterations in the expression of the epithelial marker E-cadherin and the mesenchymal marker protein were observed. Mechanistically, the Ca^2+^/CaMKII signaling pathway and autophagy were evaluated. An autophagy inhibitor 3-MA was used to examine Wnt5a in the regulation of autophagy during EMT. Furthermore, we used a CaMKII inhibitor KN-93 to determine whether Wnt5a induced autophagy overactivation and EMT *via* the Ca^2+^/CaMKII signaling pathway.

**Results:**

Asthma patients exhibited a significant increase in the gene expression of *WNT5A* compared to the healthy control. Upon HDM and IL-4 treatments, we observed that Wnt5a gene and protein expression levels were significantly increased in 16HBE cells. Interestingly, Wnt5a mimic peptide FOXY5 significantly inhibited E-cadherin and upregulated α-SMA, Collagen I, and autophagy marker proteins (Beclin1 and LC3-II). Rhodamine-phalloidin staining showed that FOXY5 resulted in a rearrangement of the cytoskeleton and an increase in the quantity of stress fibers in 16HBE cells. Importantly, blocking Wnt5a with BOX5 significantly inhibited autophagy and EMT induced by IL-4 in 16HBE cells. Mechanistically, autophagy inhibitor 3-MA and CaMKII inhibitor KN-93 reduced the EMT of 16HBE cells caused by FOXY5, as well as the increase in stress fibers, cell adhesion, and autophagy.

**Conclusion:**

This study illustrates a new link in the Wnt5a-Ca^2+^/CaMKII-autophagy axis to triggering airway remodeling. Our findings may provide novel strategies for the treatment of EMT-related diseases.

**Supplementary Information:**

The online version contains supplementary material available at 10.1186/s10020-024-00862-3.

## Introduction

Asthma is a prevalent chronic non-communicable disease, affecting approximately 334 million people worldwide. Developing countries experience an exceptionally high burden of asthma cases (Porsbjerg et al. 2023). Airway remodeling is a characteristic pathological feature of asthma, where prolonged exposure to asthmatic stimuli leads to the proliferation, differentiation, and dysfunction of airway structural cells, specifically human bronchial epithelial cells (HBECs) (Zou et al. 2019). This remodeling process involves thickening of the basement membrane, subepithelial fibrosis, epithelial detachment, and smooth muscle hypertrophy, resulting in irreversible airway stenosis and lung dysfunction in asthma patients (Hammad and Lambrecht 2021). Therefore, it is crucial to comprehend the molecular mechanism underlying asthmatic airway remodeling for effective prevention and treatment of asthma.

Fibroblasts play a significant role in airway remodeling as they are responsible for synthesizing and secreting extracellular matrix proteins. Their involvement in asthma-related airway remodeling has been extensively studied (Mostaço-Guidolin et al. 2019). Researcher suggests that epithelial-mesenchymal transition (EMT) may have a substantial impact on this process (Shi et al. 2023). Cancer cells break free from neighboring cells and the basement membrane, resulting in the spread from the primary tumor to distant organs *via* EMT (Elanany et al. 2023). EMT is more frequently observed in asthmatic HBECs (Hackett et al. 2009; Yang et al. 2017). Studies have shown that transforming growth factor-beta 1 (TGF-β1) can induce EMT in normal primary HBECs through a Smad3-dependent mechanism (Hackett et al. 2009). Additionally, house dust mites (HDM) can enhance TGF-β1-induced EMT of HBECs *via* epidermal growth factor receptors (Heijink et al. 2010). However, research also indicates that TGF-β1 inhibits the diversity of the inflammatory response in the airways. In mice exposed to ovalbumin, administration of anti-TGF-β1 antibodies can lead to increased airway hyperactivity (Alcorn et al. 2007). Therefore, a comprehensive analysis of the EMT process in airway remodeling may provide valuable insights for the development of efficient therapeutic strategies.

The Wnt signaling pathway in the lungs has attracted much attention (Ling et al. 2023). DNA microarray research has shown that patients with asthma have higher levels of Wnt5a expression in their peripheral blood, which is strongly associated with the disease (Syed et al. 2007). EMT in tumor cells has been linked to abnormalities in the Wnt5a pathway, which enhances tumor invasion and metastasis (Quezada and Lopez-Bergami 2023; Astudillo 2020; Kotrbová et al. 2020). Exogenous Wnt5a interventions, for instance, increase the expression of vimentin, snail, and other interstitial markers of melanoma cells, which promotes melanoma metastasis (Gajos-Michniewicz and Czyz 2020). While it has not been reported whether the Wnt5a signaling pathway may be involved in the EMT of HBECs during asthma.

Autophagy significantly impacts the onset of asthma, particularly in airway remodeling (McAlinden et al. 2019). Furthermore, excessive autophagy can lead to metabolic distress, destruction of cellular components, and cell death, contributing to various respiratory diseases such as acute lung injury (Yang et al. 2022), interstitial lung disease, asthma, and chronic obstructive pulmonary disease (Painter et al. 2020; Liao et al. 2019). The increased synthesis of extracellular matrix proteins during airway remodeling requires a substantial amount of energy, which can be provided by autophagy (Zeki et al. 2016). Overactive autophagy can alter the TGF-β1/Smad3 signaling pathway, promoting EMT (Chen et al. 2019). Airway mucosal biopsies from asthma patients have shown significant autophagosomes in epithelial cells and fibroblasts. Additionally, studies have demonstrated that the autophagy inhibitor chloroquine reduces airway remodeling and inflammation in asthmatic mice (McAlinden et al. 2019). It has also been reported that Wnt5a is involved in celastrol-induced autophagy in vascular smooth muscle cells (Shi et al. 2021). A higher dose of autophagy inhibitors is required to inhibit autophagy in cases of high Wnt5a expression (Ndoye et al. 2017). It has been observed that Calmodulin-dependent kinase II (CaMKII) is active in the bronchial epithelium of asthmatic patients, and the CaMKII inhibitor peptide CaMKII has shown potential in treating allergic asthma in mice (Morris et al. 2017). Furthermore, studies have shown that CaMKII can promote autophagy by phosphorylating Beclin1, an essential protein for initiating autophagy (Kang et al. 2011; Li et al. 2017).

Herein, we hypothesized that elevated Wnt5a expression in asthma could activate excessive autophagy through the Ca^2+^/CaMKII pathway, leading to the induction of the EMT process and airway remodeling, ultimately exacerbating asthma.

## Materials and methods

### Cell culture

An HBEC cell line (16HBE, ATCC) was used in this study. Cells were cultured in DMEM high-glucose medium (Hyclone, USA) containing 10% fetal bovine serum (Gibco, USA) and 1% penicillin-streptomycin mixture at 37 °C under a 5% CO_2_ atmosphere. Before treatment, cells were incubated and converged to 70-80% in the cell culture flasks.

### Cell treatment

Cells were treated as follows: HDM and IL-4: treated with 100 µg/mL HDM or 10 ng/mL IL-4 for 24 h to activate autophagy and EMT (Bai et al. 2022). FOXY5 (a Wnt5a mimic peptide): 100 µM for 24 h (Ma et al. 2023). BOX5 (a Wnt5a antagonist): Pretreated with 100 µM BOX5 for 1 h before adding IL-4 (Zou et al. 2021; Zhong et al. 2023a, b). KN-93 (a CaMKII inhibitor): 2.5 µM, added 1 h before IL-4 treatment to inhibit the CaMKII pathway (Liu et al. 2023). 3-MA (an autophagy inhibitor): 5 mM, added 1 h before IL-4 to inhibit autophagy (Pei et al. 2023).

### Western blot

Western blot was performed as previously described (Yang et al. 2020; Zhang et al. 2023). 16HBE cells were harvested and lysed in RIPA buffer (Solarbio, Beijing, China) containing protease inhibitor PMSF (Solarbio) and a phosphodiesterase inhibitor cocktail (Solarbio). Protein concentrations were determined using a BCA kit (Thermo Fisher Scientific, USA). Proteins were separated in 10% or 12% SDS-PAGE gels. Separated proteins were transferred onto polyvinylidene difluoride (PVDF) membranes, blocked with 5% skimmed milk in TBST, and incubated with the primary antibodies overnight at 4 °C. Subsequently, membranes were incubated with appropriate secondary HRP-linked antibodies. Proteins were visualized by enhanced chemiluminescence (Millipore, Burlington, MA, USA). Images were obtained using ChemiDoc XRS (Bio-Rad, Hercules, CA). The relative band intensity was quantified using the Image Lab Analyzer software (Bio-Rad). The antibodies used in the present research were as follows: rabbit anti-α-Tubulin antibody (1:2000, Servicebio, Wuhan, China); rabbit anti-α-SMA antibody (1:1000, Cell Signaling Technology, USA); rabbit anti-E-cadherin antibody (1:2000, Cell Signaling Technology); rabbit anti-Beclin1 antibody (1:1500, Cell Signaling Technology); rabbit anti-Collagen I antibody (1:1000, Cell Signaling Technology); rabbit anti-LC3 antibody (1:1500, Cell Signaling Technology); rabbit anti-Wnt5a antibody (1:1000, BOSTER, Wuhan, China); rabbit anti-CaMKII antibody (1:1000, BOSTER); rabbit anti-p-CaMKII antibody (1:1000, Cell Signaling Technology).

### RNA extraction and real-time PCR

Total RNA was extracted using RNAiso Plus (TaKaRa Clontech, Japan) according to our previously described (Zhong et al. 2019, 2023a, b). Chloroform (100 µL) was added to the extract and vortexed for 30 s. The mixtures were centrifuged at 12,000 rpm for 15 min at 4 °C, and the transparent upper layer was collected in a new tube. Each sample was mixed with isopycnic isopropanol. The samples were centrifuged at 12,000 rpm for 10 min at 4 °C. The supernatant was discarded, and the pellet was washed using 75% ethanol. The samples were centrifuged at 7,500 rpm for 5 min at 4 °C, and the supernatant was discarded. The pellet was air-dried at room temperature and dissolved in 15 µL of RNase-free water. Complementary DNA (cDNA) was generated using 1.0 µg of total RNA with the PrimeScript™RT reagent Kit with gDNA Eraser (TaKaRa Clontech). Real-time PCR analyses were performed using TB Green® Premix Ex Taq™ II reagent (TaKaRa Clontech) in triplicate on a Real-Time PCR Detection System (CFX96Touch™, Bio-Rad). All mRNA expression levels of the indicated genes were normalized against *β-ACTIN.* The sequences of the primers are shown in Table 1.

**Table 1:** Primer sequences used in this study

Gene	Forward primer (5′–3′)	Reverse primer (5′–3′)
*h-COL1A1*	CCTGGATGCCATCAAAGTCT	AATCCATCGGTCATGCTCTC
*h-VIMENTIN*	GACGCCATCAACACCGAGTT	CTTTGTCGTTGGTTAGCTGGT
*h-N-CADHERIN*	AGCCAACCTTAACTGAGGAGT	GGCAAGTTGATTGGAGGGATG
*h-WNT5A*	GCCAGTATCAATTCCGACATG	TCACCGCGTATGTGAAGGC
*h-β-ACTIN*	GGCACCCAGCACAATGAAG	CCGATCCACACGGAGTACTTG

### Wound healing assay

The 16HBE cells were cultured in a 24-well plate (5 × 10^5^ cells/well). After the cells reached 80% confluency, a scratch was created using a sterile pipette tip in the middle of each well. The cells were washed twice with phosphate-buffered saline (PBS) to smooth the scratch edge and remove floating cells. Subsequently, the cells were cultured in serum-free DMEM high-glucose medium at 37 °C with 5% CO_2_. Cell images were captured every 24 h under a fluorescence microscope at ×40 magnification. Cell migration was quantified using ImageJ analysis software 1.53 (National Institutes of Health, USA).

### Rhodamine phalloidin staining assay

The cell samples were fixed in 4% paraformaldehyde, permeabilized with 0.1% Triton X-100 for 10 min, and blocked with 5% bovine serum albumin for 1 h. Rhodamine phalloidin (Yeasen, China) was dissolved in methanol at 6.6 µM and stored at -20 °C. Before use, phalloidin was diluted (1:40) in 1% PBS. The samples were incubated with diluted phalloidin for 30 min. Nuclei were stained with 4′,6-diamidino-2-phenylindole (DAPI) by incubation for 30 s. Then, the samples were washed thrice with PBS for 5 min each. Images of F-actin were captured on a fluorescence microscope.

### Ca^2+^ level measurement

The level of intracellular Ca^2+^ was measured by Fluo-4 AM (Yeasen, China). After treatment, the 16HBE cells were incubated with 5 µM Fluo-4 AM for 30 min at 37 °C in the dark. Then, the Ca^2+^ fluorescence intensity was evaluated using the flow cytometer (Luo et al. 2020).

### Bioinformatics analysis of differentially expressed genes (DEG)

The microarray datasets of Asthma and control samples were systematically extracted from GEO (https://www.ncbi.nlm.nih.gov/geo/) (Barrett et al. 2013). Utilizing the GEO2R online analysis tool, DEGs between asthma and normal tissues or cells were identified. Upon accessing the relevant GEO data website, the built-in GEO2R link was selected, followed by configuring groups by clicking on “Define Groups.” Subsequently, all asthma samples within the dataset were assigned to the experimental group, while all adjacent normal samples were designated as the control group. After maintaining other settings as default configurations on the website, online analysis was performed, and all analysis results were exported in Excel format. In the resultant dataset, genes meeting the cutoff criterion of the adjusted *p*-value < 0.05 and |log_2_FC| > 1 were considered DEGs. Finally, the target genes were extracted for statistical analysis.

### Statistical analysis

All quantitative measurements were done at least in triplicate, and mean ± SD was presented. Comparison of the mean values between different groups was performed by one-way analysis of ANOVA test (Dunnett’s test) or two-tailed unpaired *t*-test using GraphPad software 9.0.1 (San Diego, CA, USA). A *P*-value ≤ 0.05 was considered to be statistically significant.

## Results

### WNT5A (Wnt5a) are highly enriched in the HBECs from asthma patients, lung of asthma mice, and HDM or IL-4-treated HBECs

**Fig. 1:**
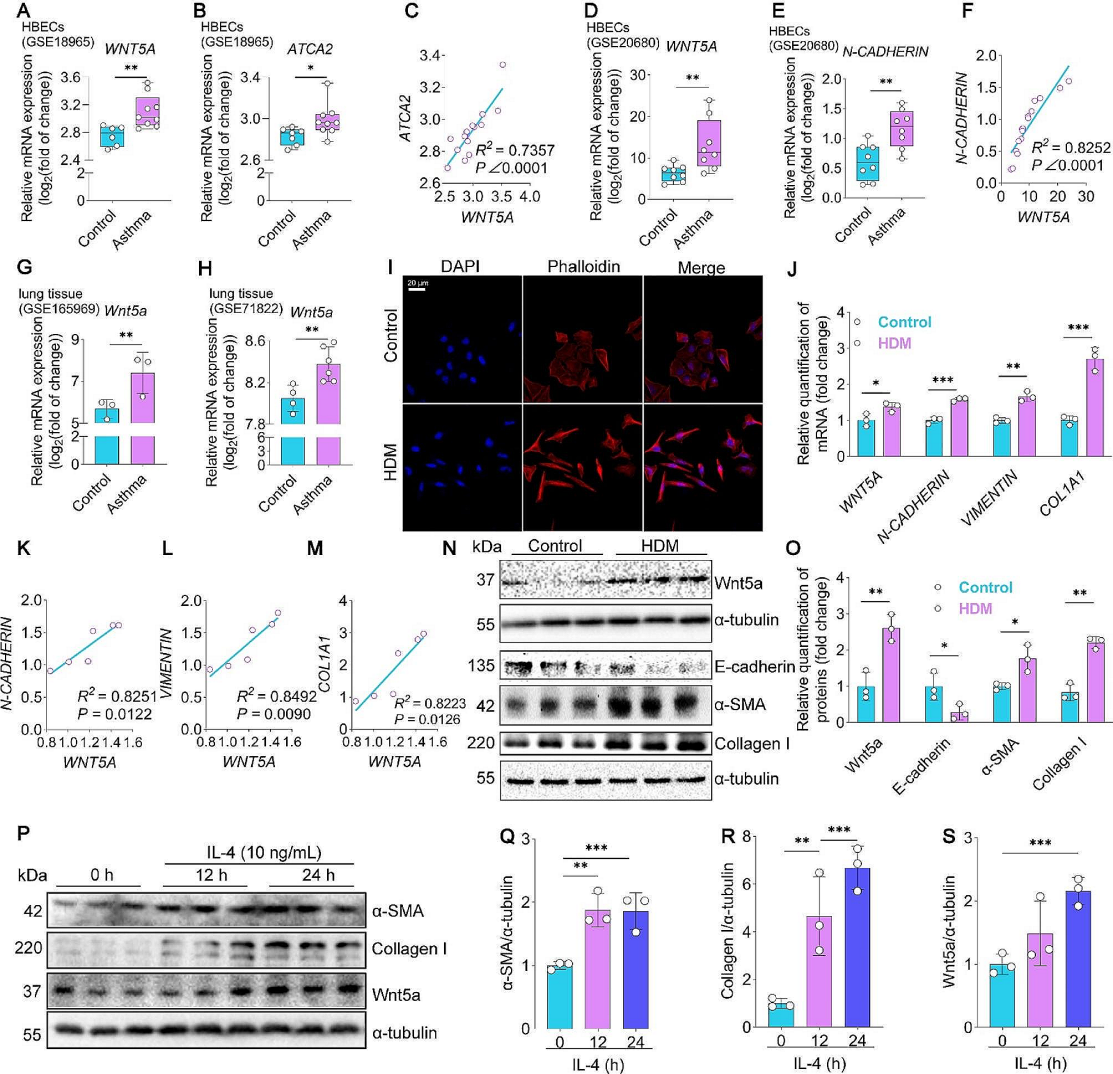
HDM or IL-4 treatment increases Wnt5a and promotes EMT in 16HBE cells. (**A-C**) Microarray analysis examines patterns and correlations of *WNT5A* and *ACTA2* in HBECs derived from asthma patients, utilizing data from the GSE18965 dataset, *n* = 9. (**D-F**) Microarray analysis scrutinizes expression patterns and conducts correlation analysis of *WNT5A* and *N-CADHERIN* in HBECs obtained from asthma patients, using data from the GSE20680 dataset, *n* = 9. (**G-H**) Microarray analysis was conducted to examine the expression of WNT5A in mouse asthmatic lung tissues from the GSE165969 and GSE71822 datasets, *n* = 3–6. (**I**) Rhodamine phalloidin staining was used to determine HDM-induced cytoskeleton morphology in 16HBE cells (bar = 20 μm). (**J-M**) Real-time PCR was employed to assess the mRNA expression levels of *WNT5A, VIMENTIN, N-CADHERIN*, and *COL1A1* in addition to conducting a correlation analysis between EMT-related genes and *Wnt5a* in 16HBE cells subjected to HDM treatment. Data were normalized to *β-ACTIN*, *n* = 3. (**D-E**) The protein level of Wnt5a was detected by western blot, *n* = 3. (**N-O**) Western blot and quantification of E-cadherin, α-SMA, and Collagen I were performed on 16HBE cells exposed to HDM, *n* = 3. (**P-S**) Western blot and quantification of α-SMA, Collagen I, and Wnt5a were conducted on 16HBE cells treated with IL-4, *n* = 3. Bar graphs represent mean ± SD. *P < 0.05, **P < 0.01, and ***P < 0.001

To investigate the role of Wnt5a in the pathology of asthma, we first interrogated the transcript levels of WNT5A (Wnt5a) in the human bronchial epithelial cells (HBECs) from patients with asthma and lung from mice with asthma by analyzing the GEO datasets of human (GSE18965 (Kicic et al. 2010), GSE206680 (Murphy et al. 2023),), and GEO data of mouse (GSE165969 (Jaiswal et al. 2022), and GSE71822 (Eyring et al. 2018). Impressively, we found that WNT5A was highly enriched in HBECs from patients with asthma (Fig. 1A, D**)**. Next, we assessed the relationship between Wnt5a and EMT in HBECs from asthma patients. As shown in Fig. 1B, E, mesenchymal cell markers alpha 2 smooth muscle actin (ACTA2) and N-CADHERIN were significantly upregulated in HBECs. Interestingly, the level of WNT5A expression was positively correlated with ATCA2 and N-CADHERIN mRNA expression (Fig. 1C, F**)**. Consistently, Wnt5a levels were elevated in the lung of asthma mice, compared to controls (Fig. 1G-H**)**. These data suggest that WNT5A may play a critical role in EMT during asthma. We then focused on HBECs and further determined the expression of WNT5A in HBECs in response to HDM in vitro. To generate an EMT model in vitro, 16HBE cells were treated with HDM. The actin cytoskeleton was visualized using phalloidin. As shown in Fig. 1I, HDM-treated 16HBE cells showed remodeled cytoskeletons, increased stress fibers, and intercellular adhesion. In addition, we determined mRNA levels of mesenchymal markers and WNT5A in these HDM-treated 16HBE cells using RT-qPCR. Our results showed that *WNT5A* and mesenchymal markers *N-CADHERIN*, *VIMENTIN*, and *COL1A1* mRNA levels significantly increased compared to the control (Fig. 1J). Importantly, *WNT5A* expression was positively correlated with *N-CADHERIN* (Fig. 1K), *VINMENTIN* (Fig. 1L), and *COL1A1* (Fig. 1M) mRNA expression. In parallel, the protein of Wnt5a and mesenchymal markers (α-SMA and Collagen I) were significantly increased, while the epithelial marker E-Cadherin was markedly decreased (Fig. 1N-O). Similar results were also observed in 16HBE cells challenged with IL-4, as indicated by significantly upregulated α-SMA, Collagen I, and Wnt5a (Fig. 1P-S). Together, both in vivo and in vitro results consistently indicate that the expression of WNT5A is enriched during asthma, and WNT5A may be strongly correlated with EMT.

### Wnt5a activation triggers EMT in vitro

**Fig. 2:**
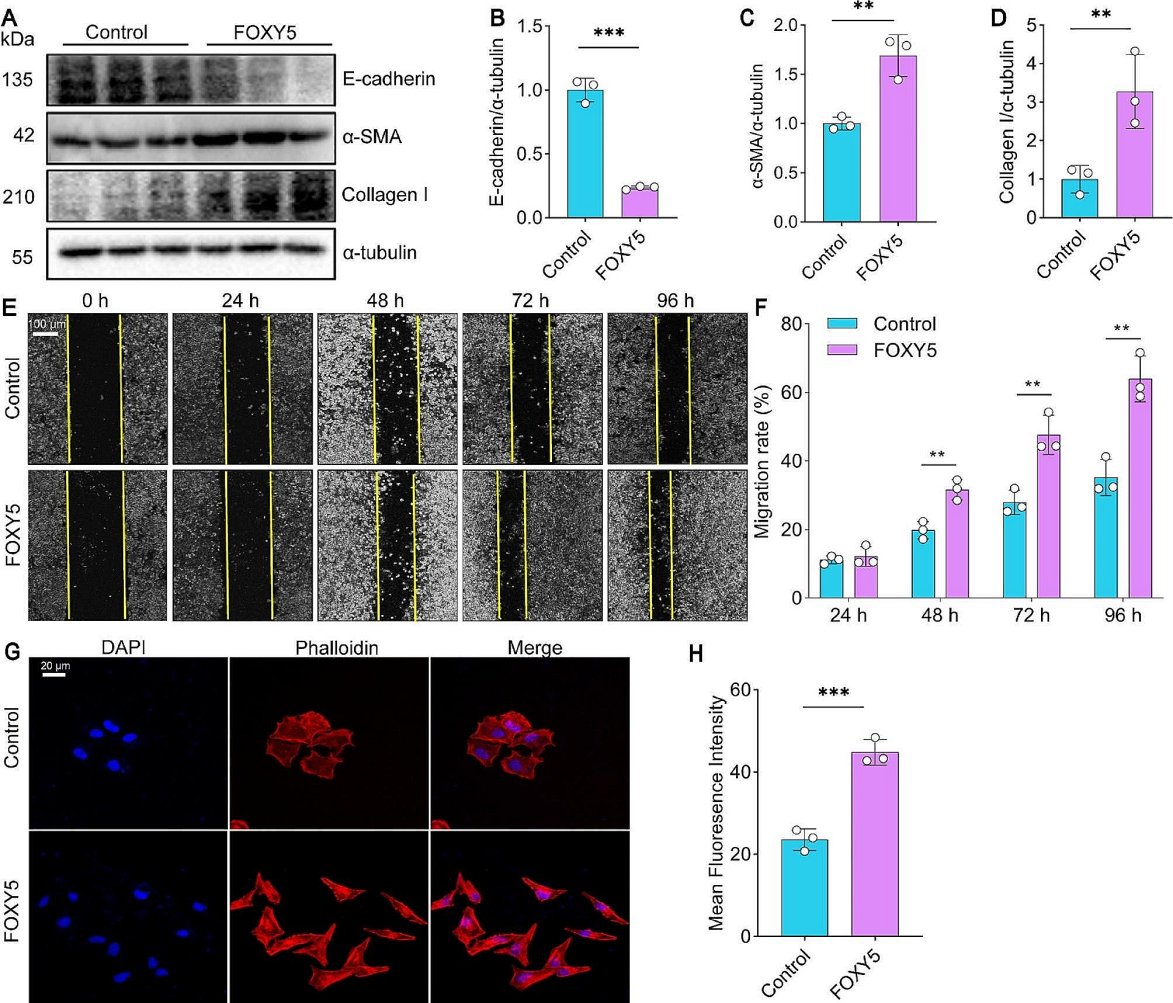
FOXY5 promotes EMT in 16HBE cells. (**A-D**) Western blot was used to measure and quantify the E-cadherin, α-SMA, and Collagen I protein levels in 16HBE cells exposed to FOXY5, *n* = 3. (**E**) Migration of 16HBE cells was observed by wound healing assay after FOXY5 treatment. (**F**) Mean percentage of cells migrating in the wound healing assay, *n* = 3. (**G-H**) Rhodamine phalloidin staining and quantitative analysis were employed in 16HBE cells to investigate the cytoskeletal morphology modifications induced by FOXY5. Bar graphs represent mean ± SD. **P < 0.01 and ***P < 0.001

To investigate the direct effect of Wnt5a on EMT, we utilized FOXY5, an analog peptide for Wnt5a, to treat 16HBE cells. We found that the protein expression of E-cadherin was significantly decreased, while the expression of Collagen I and α-SMA was increased (Fig. 2A-D). More interestingly, activation of Wnt5a with FOXY5 significantly increased the migration of 16HBE cells, as evidenced by the wound healing assay (Fig. 2E-F). In addition, results of Rhodamine phalloidin staining demonstrated that FOXY5 triggered alterations in the cytoskeleton, an enlargement of the F-actin of stress fibers, and increased intercellular adhesion in 16HBE cells (Fig. 2G-I). Put together, these in vitro data suggest that Wnt5a activation triggers EMT in HBECs.

### Wnt5a blockade inhibits IL-4-induced EMT in vitro

**Fig. 3:**
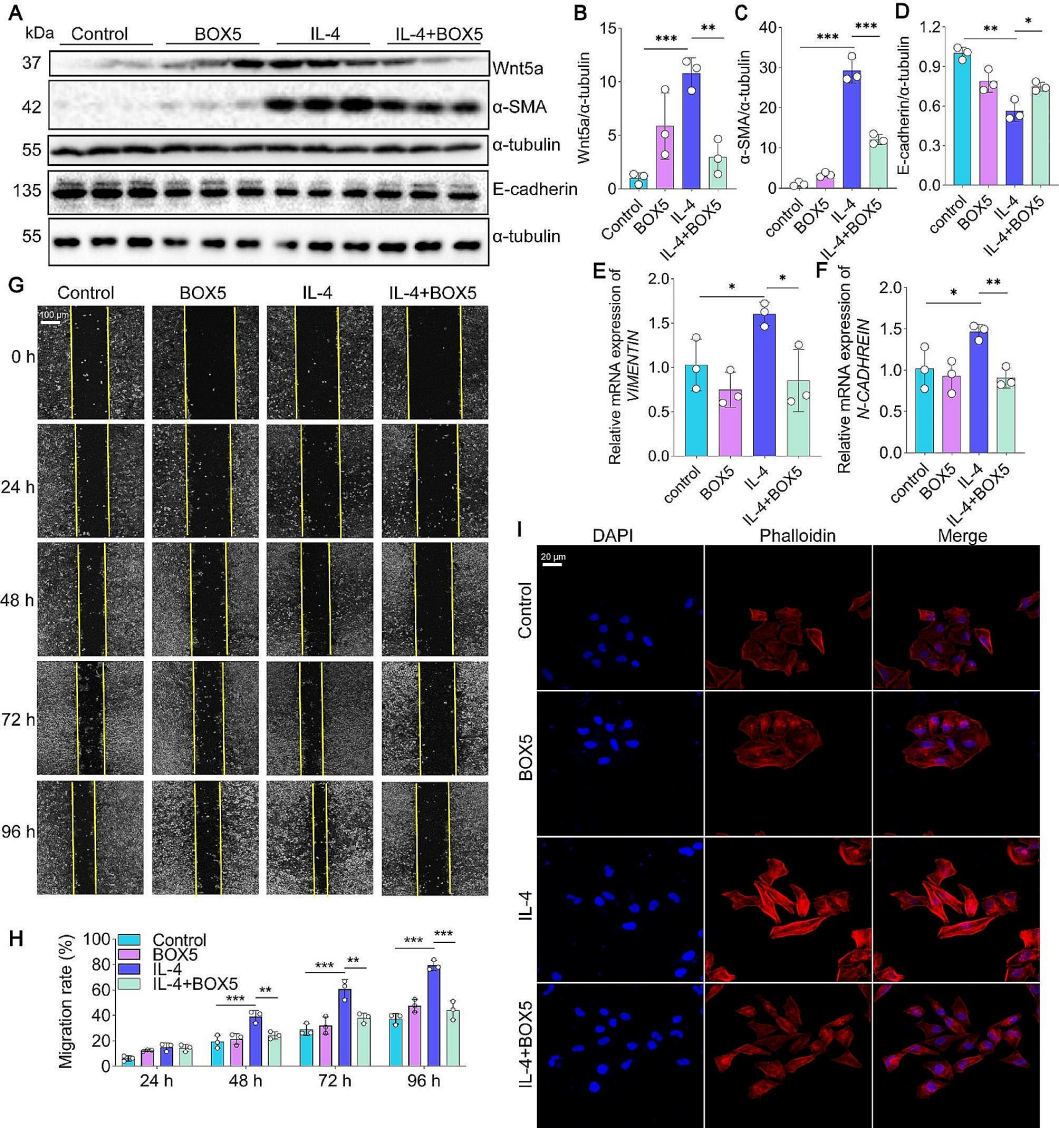
BOX5 alleviates IL-4-induced EMT in 16HBE cells. (**A-D**) Western blot and quantification were employed to measure Wnt5a, α-SMA, and E-cadherin, *n* = 3. (**E-F**) Real-time PCR was employed to assess the mRNA expression levels of *VIMENTIN* and *N-CADHERIN* in 16HBE cells subjected to IL-4 after the BOX5 treatment. Data were normalized to *β-ACTIN*, *n* = 3. (**G**) The wound healing assay was utilized to observe the migration of IL-4-treated 16HBE cells at different times after BOX5 intervention, and (**H**) migration rate was analyzed, *n* = 3. (**I**) Rhodamine phalloidin staining for IL-4-induced cytoskeleton morphology and BOX5 intervention. Data are shown as the mean ± SD. **P < 0.01 and ***P < 0.001

Next, we examined whether the blockade of Wnt5a could inhibit IL-4-induced EMT in vitro. BOX5, a specific Wnt5a antagonist derived from Wnt5a-derived hexapeptide (Jain et al. 2022), was used to block Wnt5a. Encouragingly, the results showed that the expression of Wnt5a and α-SMA protein in 16HBE cells in the IL-4 + BOX5 group was downregulated compared with that in the IL-4 group (Fig. 3A-D). Our results showed that mesenchymal markers *N-CADHERIN* and *VIMENTIN* mRNA levels were significantly decreased compared to the IL-4 group (Fig. 3E-F). Furthermore, the wound healing assay results revealed that BOX5 significantly reduced the migration of IL-4-induced 16HBE cells (Fig. 3G-H). Rhodamine phalloidin staining also exhibited that BOX5 effectively inhibited the IL-4-induced remodeling of the cytoskeleton in 16HBE cells, as evidenced by the reduction in F-actin stress fibers and intercellular adhesion (Fig. 3I). These findings strongly suggest that blocking Wnt5a can impede EMT in 16HBE cells induced by IL-4.

### Excessive autophagy is required for Wnt5a-induced EMT in vitro

**Fig. 4:**
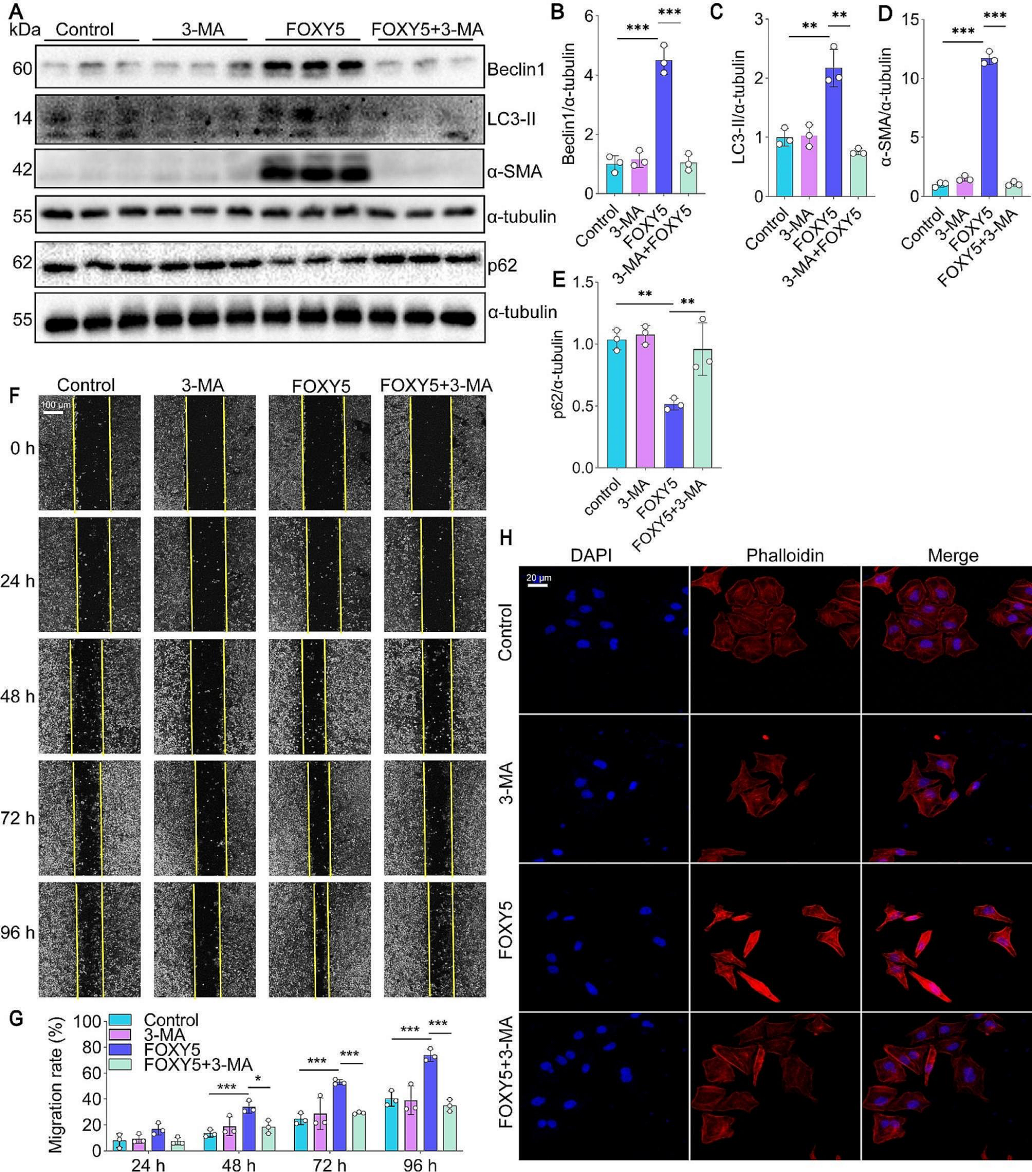
3-MA relieves FOXY5-induced EMT and autophagy of 16HBE. (**A-E**) Western blot and quantification of α-SMA, Beclin1, LC3-II, and p62 were performed, *n* = 3. (**F-G**) The wound healing assay was employed to assess the migration of 16HBE cells induced by FOXY5 after the 3-MA intervention, *n* = 3. (**H**) Rhodamine phalloidin staining for FOXY5-induced cytoskeleton morphology and 3-MA intervention. Data are shown as the mean ± SD. *P < 0.05, **P < 0.01, and ***P < 0.001

Considering that excessive autophagy has been shown to promote the occurrence and development of EMT (Liu et al. 2017). Interestingly, we observed that FOXY5 significantly increased the protein levels of autophagy markers Beclin1 and LC3-II while downregulating the level of p62 (Fig. 4A-C, E). To investigate the role of autophagy in Wnt5a-induced EMT in HBECs, we pretreated 16HBE cells with 3-MA, an autophagy inhibitor, before administering FOXY5. Our results demonstrated that 3-MA had a notable effect on reducing the expression of the EMT-related marker α-SMA protein, as well as the autophagy-related markers LC3-II and Beclin1 in FOXY5-treated 16HBE cells (Fig. 4A, D). Furthermore, in a wound healing assay, 3-MA treatment led to a significant decrease in the migration of 16HBE cells stimulated by FOXY5 (Fig. 4F-G). Additionally, Rhodamine-phalloidin staining revealed that 3-MA treatment resulted in reduced cytoskeletal remodeling, decreased stress fiber proliferation, and increased intercellular adhesion in FOXY5-treated 16HBE cells (Fig. 4I). Taken together, these findings suggest that excessive autophagy is required for Wnt5a-induced EMT in 16HBE cells.

### Wnt5a induces excessive autophagy *via* Ca^2+^/CaMKII

**Fig. 5:**
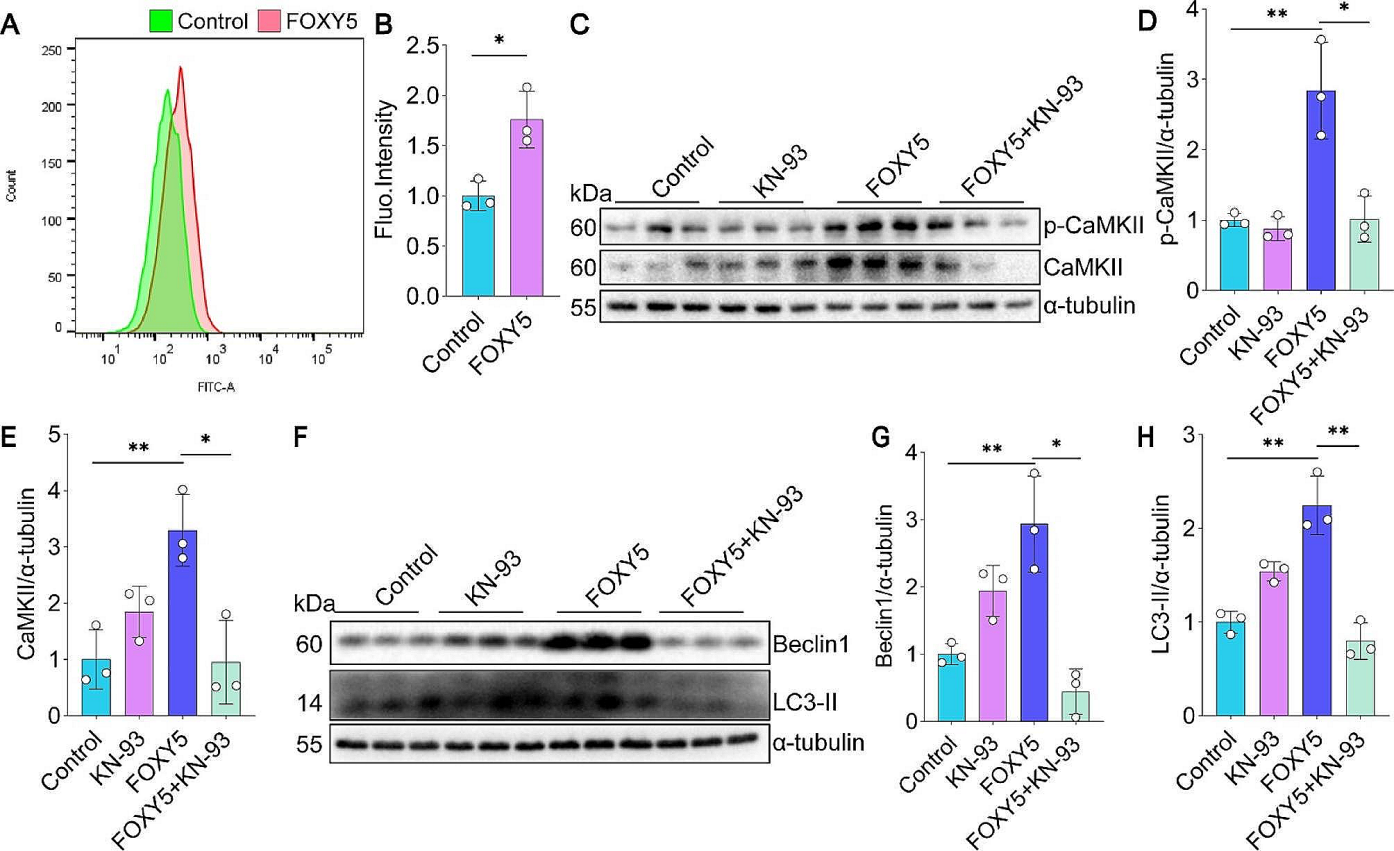
Blockade of CaMKII inhibits FOXY5-induced autophagy in 16HBE cells. (**A-B**) A flow cytometer was employed to measure the content of Ca^2+^ in 16HBE cells exposed to FOXY5. (**C-E**) Western blot detected the expression of CaMKII and phospho-CaMKII proteins in 16HBE cells treated with/without FOXY5 and with/without KN-93, *n* = 3. (**F-H**) Western blot and quantitation of Beclin1 and LC3-II protein expression were performed in 16HBE cells, *n* = 3. Data are shown as the mean ± SD. *P < 0.05 and **P < 0.01

Then, we wondered how Wnt5a induced excessive autophagy to promote EMT in HBECs. Given that autophagy can be activated through the phosphorylation of Beclin1 by CaMKII More importantly, FOXY5 significantly increased the level of Ca^2+^ compared to the control group (Fig. 5A-B). Additionally, FOXY5 significantly increased the p-CaMKII and CaMKII levels in 16HBE cells (Fig. 5C-E). To further clarify the role of Ca^2+^/CaMKII in excessive autophagy regulation in 16HBE cells, we utilized a specific inhibitor called KN-93. Interestingly, we observed that KN-93 effectively reduced the expression and phosphorylation level of CaMKII in FOXY5-treated 16HBE cells (Fig. 5C-E), along with decreased expression of autophagy-related markers, such as Beclin1 and LC3-II (Fig. 5F-H). These results suggest that Wnt5a induces excessive autophagy in 16HBE cells by activating the Ca^2+^/CaMKII pathway. We also investigated whether blocking Wnt5a could inhibit IL-4-induced activation of the autophagy pathway and CaMKII signaling pathway in vitro. The results indicated that, compared to the IL-4 group, the IL-4 + BOX5 group exhibited downregulation of autophagy-related proteins LC3 and Beclin1, as well as a decrease in CaMKII activity in 16HBE cells (Supplementary Figure S1).

### Wnt5a promotes EMT in 16HBE cells through Ca^2+^/CaMKII

**Fig. 6:**
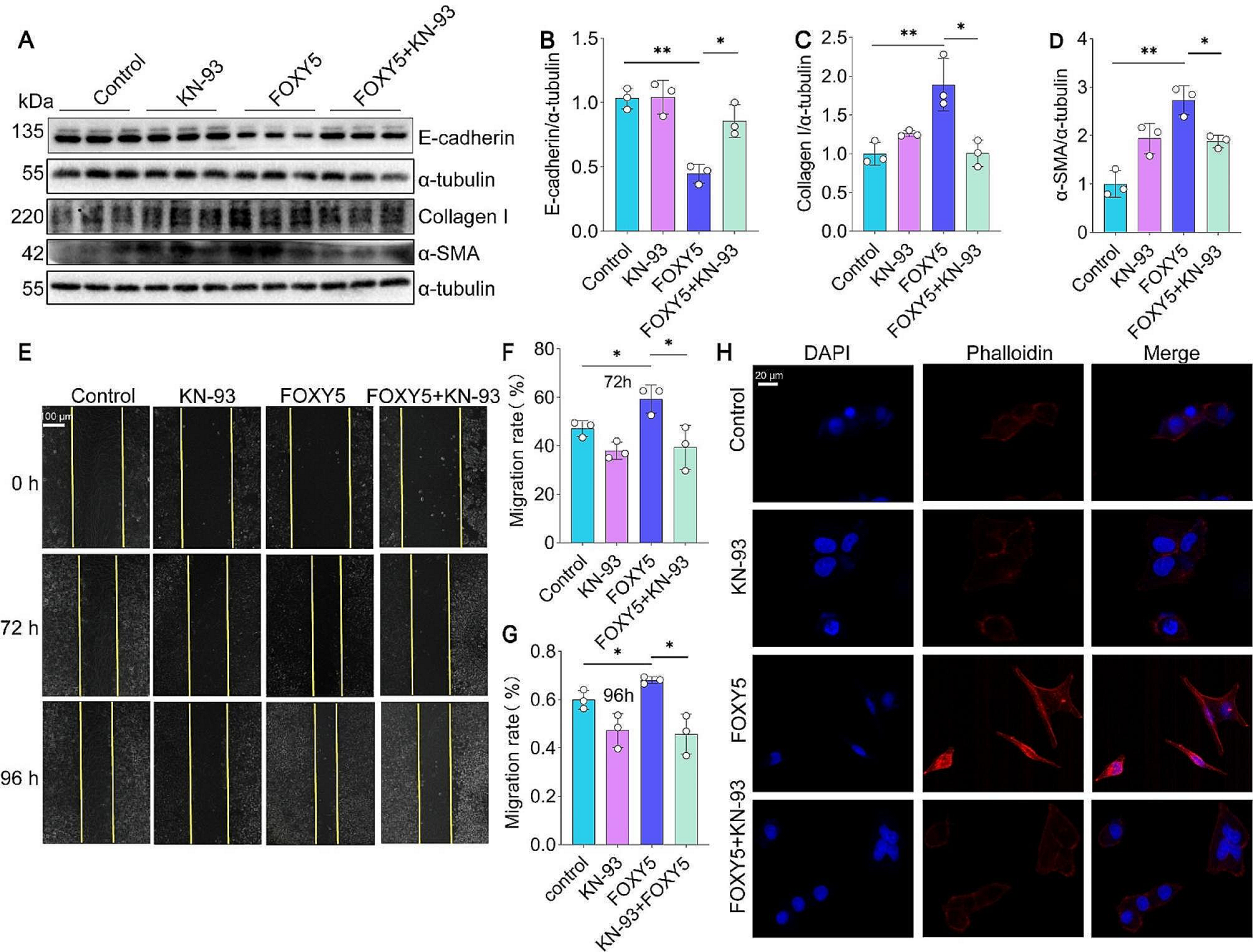
Blockade of CaMKII inhibits FOXY5-induced EMT in 16HBE cells. (**A-D**) Western blot and quantification of E-cadherin, α-SMA, and Collagen I were detected, *n* = 3. (**E-G**) Migration and quantification of 16HBE cells were observed by wound healing assay, *n* = 3. (**H**) Rhodamine phalloidin staining was implemented in 16HBE cells with KN-93 intervention. Data are shown as the mean ± SD. *P < 0.05 and **P < 0.01

Lastly, we investigated whether the Wnt5a-activated Ca^2+^/CaMKII pathway promoted EMT in HBECs. We found that KN-93 significantly reduced the expression of EMT-related markers, including α-SMA and Collagen I protein (Fig. 6A-D). Additionally, the intervention of KN-93 resulted in a notable reduction in FOXY5-induced cell migration in 16HBE cells, as observed through the wound healing assay (Fig. 6E-G). Moreover, the staining of Rhodamine phalloidin demonstrated that KN-93 effectively reduced the cytoskeleton changes and stress fiber proliferation induced by FOXY5 in 16HBE cells (Fig. 6H). Collectively, these results indicate that the Ca^2+^/CaMKII pathway mediates FOXY5-induced EMT in 16HBE cells.

## Discussion

**Fig. 7:**
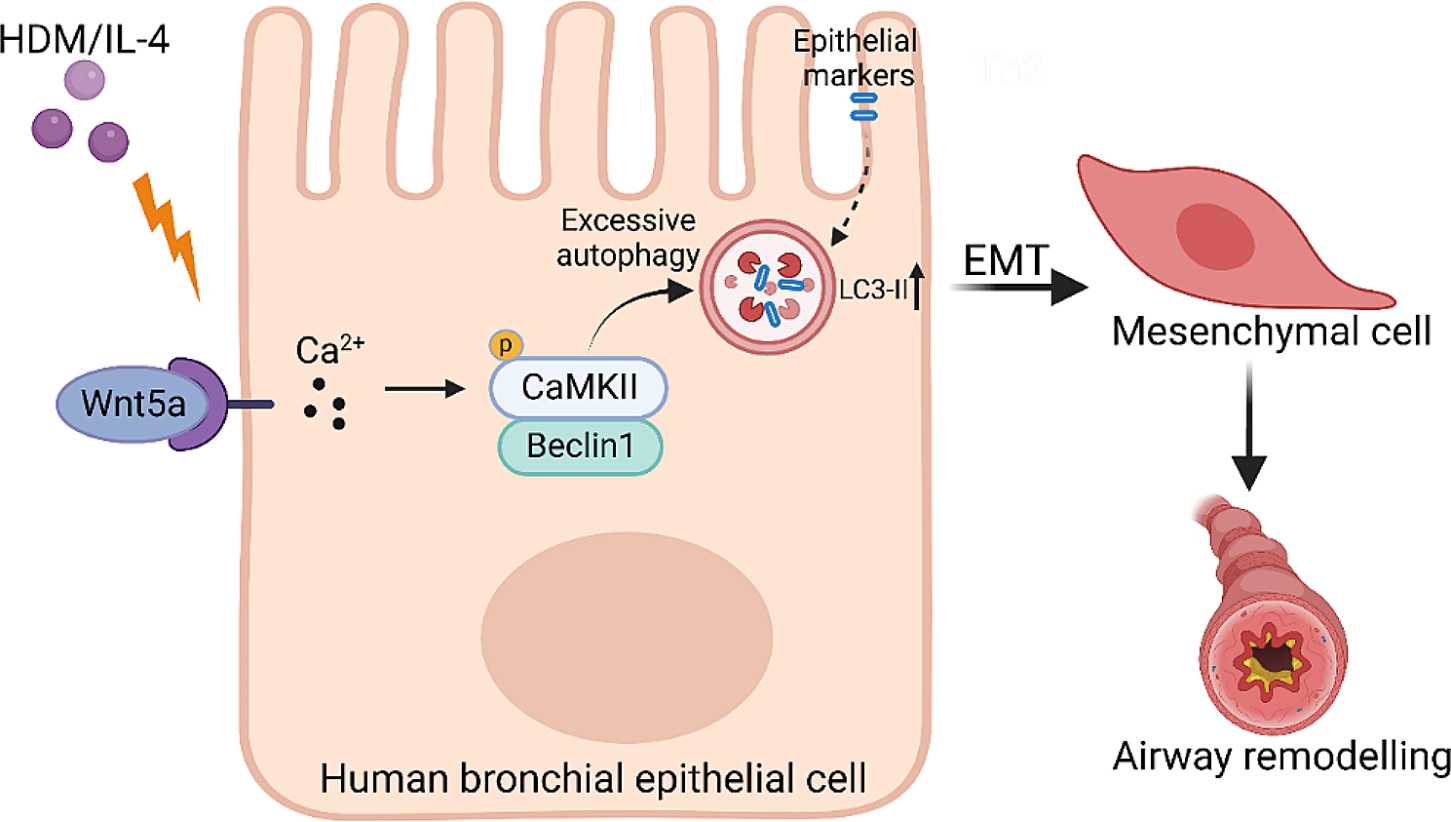
Schematic illustration of the mechanisms of Wnt5a-induced EMT in HBECs. This hypothesis illustrates a molecular pathway in HBECs leading to airway remodeling, typically associated with classical autophagy pathway and EMT. In short, Wnt5a induced by HDM or IL-4 leads to the overactivation of autophagy in HBECs through the Ca^2+^/CaMKII signaling pathway, subsequently promoting bronchial epithelial EMT and triggering airway remodeling

In this study, for the first time, we demonstrate that Wnt5a is a novel modulator of EMT in HBECs during asthma. Using GEO datasets and in vitro cultured HBECs, we uncovered that WNT5A (Wnt5a) is highly enriched in the HBECs from asthma patients, lung of asthma mice, and HDM or IL-4-treated HBECs. Wnt5a mimic peptide FOXY5 significantly inhibited E-cadherin but upregulated α-SMA and Collagen I, triggered rearrangement of the cytoskeleton, and increased the number of stress fibers in HBECs. By contrast, blocking Wnt5a with BOX5 significantly inhibited EMT induced by IL-4 in HBECs. Mechanistically, active Wnt5a triggered the activation of Ca^2+^/CaMKII, leading to excessive autophagy. Moreover, CaMKII inhibitor KN-93 and autophagy inhibitor 3-MA could reduce the FOXY5-induced EMT, the increased stress fibers, and the cell adhesion (Fig. 7). The study suggests that Wnt5a-mediated activation of the Ca^2+^/CaMKII axis could be a novel target for the treatment of EMT during asthma.

Airway remodeling is a complex process involving various cellular changes, such as harm to the epithelium, fibrosis beneath the epithelium, increased airway smooth muscle, hypertrophied and proliferated goblet cells, and angiogenesis. This intricate process plays a significant role in the accelerated loss of lung capacity (Durrani et al. 2011). In asthma, airway remodeling requires EMT, and exposure to HDM extract leads to increased expression of IL-33 in alveolar epithelial cells. This, in turn, stimulates CD146 expression, which contributes to the remodeling of the airways during chronic allergic inflammation (Sun et al. 2020). In this study, HDM-exposed HBECs showed significantly increased expression of Wnt5a. Furthermore, analysis of transcriptome data from asthma patients’ HBECs revealed elevated Wnt5a gene expression. The Th2 cytokine IL-4, known for its pro-inflammatory and pro-remodeling effects in asthmatic airway tissue, can mimic the chronic inflammation and tissue remodeling induced by the internal environment of tissue cells (Massey and Suphioglu 2021; Oh et al. 2010). The same results were obtained after inducing IL-4, proving that Wnt5a was overexpressed in HBECs during asthma. According to the report, asthmatic patients have significantly higher Wnt5a expression in their airway smooth muscle cells, and TGF-β1-induced extracellular matrix synthesis is thought to be mediated through the Wnt5a-mediated signaling pathway (Kumawat et al. 2013). To investigate whether Wnt5a directly promotes EMT in HBECs, we treated 16HBE cells with an exogenous Wnt5a mimic peptide, FOXY5. The expression of E-cadherin, a protein associated with epithelial cells, was reduced, while the expression of α-SMA, an interstitial marker, and the extracellular matrix component Collagen I were increased. This resulted in alterations in the cytoskeleton, including increased stress fibers, decreased intercellular adhesion, and enhanced cell migration. The results demonstrate that Wnt5a activation can induce EMT in HBECs. Moreover, the application of Wnt5a antagonist to 16HBE cells significantly impeded EMT.

In this study, we have discovered a novel mechanism through which Wnt5a induces EMT in HBECs. Autophagy, a metabolic system that helps cells survive by breaking down cellular components and providing raw materials for new molecule production, plays a crucial role in this process (Vargas et al. 2023). Autophagy supplies the energy required for modifying cell structure and generating numerous interstitial proteins, supporting the biological activity involved (Zeki et al. 2016; Hernandez-Gea et al. 2012). Moreover, autophagy is closely linked to the phenotypic transformation of cells as it regulates structural proteins and regulatory components necessary for maintaining epithelial properties. For instance, autophagy is induced by water stress proteins, which suppress the PI3K/AKT/mTOR pathway, leading to the inhibition of EMT in DLD-1 cells (Guo et al. 2018). Additionally, Sphingosine kinase 1 mediates Beclin1-induced autophagy and lysosomal degradation of E-cadherin, promoting EMT in HepG2 hepatoma cells (Liu et al. 2017). Our findings demonstrate that Wnt5a significantly upregulates the expression of LC3-II and Beclin1 in 16HBE cells. Furthermore, the effects of Wnt5a-induced autophagy and α-SMA expression were significantly attenuated by the use of 3-MA, suggesting that Wnt5a induces EMT in HBECs through autophagy. Studies have shown that the Ca^2+^/CaMKII pathway is activated in the bronchial epithelium of asthma patients CaMKII has been found to directly phosphorylate Beclin1 at Ser90, leading to enhanced K63 ubiquitination and activation of autophagy Importantly, we observed that Wnt5a-mediated autophagy and EMT were reduced when a CaMKII inhibitor was applied to block the Ca^2+^/CaMKII signaling pathway, indicating that Wnt5a relies on Ca^2+^/CaMKII to induce autophagy and activate EMT.

Wnt5a has been widely recognized as an essential factor in tissue repair and regeneration. Furthermore, The involvement of Wnt5a in regulation of cancer cell invasion, metastasis, metabolism and inflammation renders it a subject of intense research in oncology (Liu et al. 2024). Data also show that Box5 pretreatment inhibited the expression of PLC, p-CaMKII, p65 and attenuated the injury of renal tubular epithelial cells and suppressed the upregulated expression of tissue remodeling cytokines (Zuo et al. 2022). Recent studies demonstrated that chemically conjugating the Foxy5 peptide to the biomaterial scaffold can more effectively activate non-canonical Wnt signaling and promote osteogenic differentiation of seeded stem cells (Deng et al. 2022). This suggests that Wnt5a-based interventions, when combined with novel biomaterials, can be applied in clinical treatments, representing an exciting scientific advancement. The mechanistic insights gathered from clinical studies of patients, as well as the application of Wnt5a intervenor, hold significant clinical implications for the development of therapeutic approaches aimed at preventing and treating various mesenchymal transition and repair dysregulation diseases, including asthma.

The study’s novelty lies in identifying the Wnt5a-mediated Ca^2+^/CaMKII axis as a potential therapeutic target for treating EMT in asthma, offering new avenues for research and treatment strategies in managing asthma-related airway remodeling. The present study has some limitations that should be addressed in future research. Our findings indicate that Wnt5a can enhance autophagy in HBECs. Further studies are required to confirm whether CaMKII promotes autophagy through Beclin1 phosphorylation. Additionally, while we have observed that inhibiting autophagy significantly reduces EMT in HBECs, the mechanism by which autophagy promotes EMT needs to be investigated in future studies. Our study fails to address non-coding RNAs, such as microRNAs and long non-coding RNAs, in relation to autophagy and Wnt signaling (Mahmoud et al. 2021). However, these non-coding RNAs might serve as potential targets for preventing Wnt signaling pathway activation. It is also important to note that we have not confirmed the change in bronchial epithelium Wnt5a in the animal model of asthma. Our observations were limited to its increase in HDM or IL-4-induced 16HBE cells.

In general, following the occurrence of a disease, resorting to various therapeutic measures to alleviate symptoms represents a measure of last resort. Conversely, the judicious selection of pharmaceuticals and administration of various vitamins, such as Vitamin E, as prophylactic agents in disease management constitute a superior approach (Hamdy et al. 2009). Respiratory diseases pose a grave threat to human life. Future research endeavors necessitate continuous assessment of both the therapeutic efficacy and adverse effects of pharmaceuticals, thereby enabling the selection of rational preventive and therapeutic strategies tailored to various diseases.

## Conclusion

In summary, the results of this study suggest that Wnt5a can lead to excessive autophagy in HBECs, which triggers EMT *via* the Ca^2+^/CaMKII pathway. Asthma medication that targets Wnt5a may be effective, opening up new possibilities for the diagnosis and management of this condition.

### Electronic supplementary material

Below is the link to the electronic supplementary material.

Supplementary Material 1

## Data Availability

The datasets analyzed during the current study are available in the GEO (ncbi.nlm.nih.gov/geo) repository. GEO belongs to the public database, and the patients involved in the database have obtained ethical approval. GEO allows users to download relevant data for free for research and publish relevant articles. The data that support the findings of this study are available from the corresponding author upon reasonable request.

## Abbreviations

AKT: Protein kinase B
CaMKII: Calmodulin-dependent kinase II
DEG: Differentially expressed genes
EMT: Epithelial-mesenchymal transition
GEO: Gene Expression Omnibus database
HBECs: Human bronchial epithelial cells
HDM: House dust mites
IL-4: Interleukin-4
IL-33: Interleukin-33
mTOR: Mammalian target of rapamycin
PI3K: Phosphatidylinositol-3-kinase
TGF-β1: Transforming growth factor-beta 1
WNT5A: Wingless-type MMTV integration site family, member 5 A
