# Supplementary material for: Wnt5a-mediated autophagy contributes to the epithelial-mesenchymal transition of human bronchial epithelial cells during asthma
Source: Mol Med. 2024 Jun 19;30:93. doi: 10.1186/s10020-024-00862-3 (PMC11188189; doi:10.1186/s10020-024-00862-3)
Supplement: Supplementary file 1 — Supplementary Material 1 [file 10020_2024_862_MOESM1_ESM.docx]

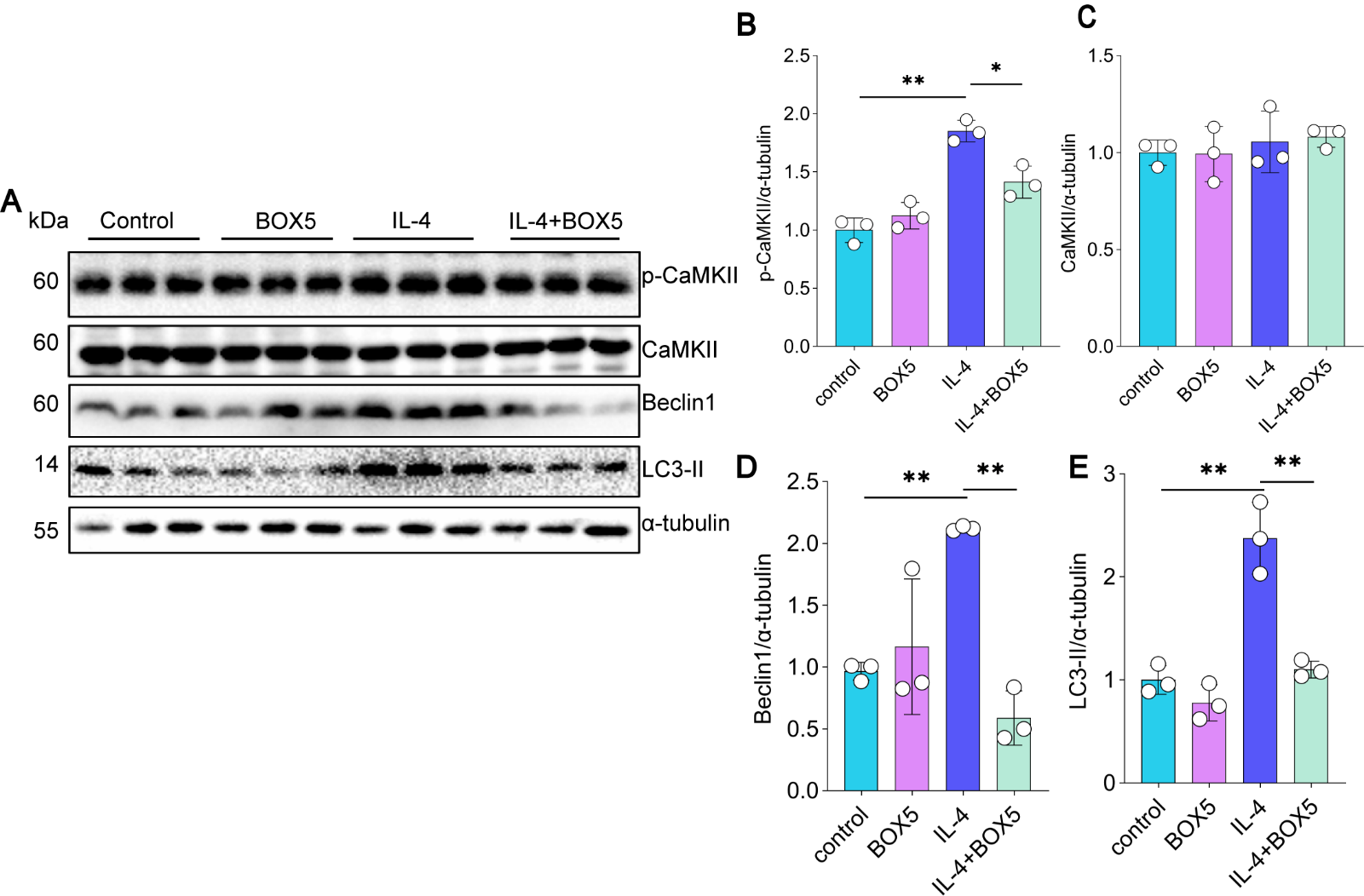


**Supplementary Figure S1. Blockade of Wnt5a inhibits IL-4-induced CaMKII activation and autophagy in 16HBE cells.** (A-E) Western blot and quantification were employed to measure CaMKII, phospho-CaMKII, Beclin1, and LC3-II proteins, *n*=3. Data are shown as the mean ± SD. **P*< 0.05 and ***P*< 0.01.
